# Supplementary material for: One-dimensional single atom arrays on ferroelectric nanosheets for enhanced CO2 photoreduction
Source: Nat Commun. 2024 Jan 5;15:305. doi: 10.1038/s41467-023-44493-4 (PMC10770382; doi:10.1038/s41467-023-44493-4)
Supplement: Supplementary file 1 — Supplementary Information [file 41467_2023_44493_MOESM1_ESM.pdf]

Supplementary Information

One-dimensional single atom arrays on ferroelectric nanosheets for enhanced CO_2 photoreduction

Lizhen Liu[1,2,†], Jingcong Hu[3,†], Zhaoyu Ma[4], Zijian Zhu[1], Bin He[1], Fang Chen[1,*], Yue Lu[3,*], Rong Xu[2], Yihe Zhang[1], Tianyi Ma[5], Manling Sui[3] and Hongwei Huang[1,*]

[1]Engineering Research Center of Ministry of Education for Geological Carbon Storage and Low Carbon Utilization of Resources, Beijing Key Laboratory of Materials Utilization of Nonmetallic Minerals and Solid Wastes, National Laboratory of Mineral Materials, School of Materials Science and Technology, China University of Geosciences (Beijing), Beijing 100083, China

[2]School of Chemistry, Chemical Engineering and Biotechnology, Nanyang Technological University, Singapore 637459, Singapore

[3]Beijing Key Laboratory of Microstructure and Properties of Solids, Faculty of Materials and Manufacturing, Beijing University of Technology, Beijing 100124, China

[4]School of Physics, Beihang University, Beijing 100191, China

[5]School of Science, RMIT University, Melbourne, VIC 3000, Australia

[†]These authors contribute equally to this article.

*Corresponding author: chenfang@cugb.edu.cn (F. Chen); luyue@bjut.edu.cn (Y. Lu); hhw@cugb.edu.cn (H.W. Huang)

Table of Contents

Supplementary Figures **3**

1. XRD patterns of the as-synthesized products 3

2. SEM images of the as-synthesized products 4

3. AFM image of BTOAuNP 5

4. TEM images of BTOAuNP 6

5. HAADF-STEM images and HAADF intensity profiles of $BTOAu_3$ and BTOPAu 7

6. EXAFS data fitting analysis 8

7. XPS spectra of the as-synthesized products 9

8. Raman spectra of the as-synthesized products 10

9. HAADF-STEM images of metals loaded on BTO and BTOP 11

10. CO production over BTOAuNP and BTOPAuNP. 13

11. Cycling test 14

12. Mott-Schottky plots of as-synthesized products 15

13. DRS spectra and energy band diagram of as-synthesized products 16

14. PL spectra of as-synthesized products 17

15. Surface charge density of BTO and BTOPAu 18

16. Relax structures of DFT calculation 19

Supplementary Tables **24**

Supplementary Table 1. Au content based on ICP results. 24

Supplementary Table 2. EXAFS fitting parameters at the Au L_3-edge for samples. 25

Supplementary Table 3. Comparison of the CO_2 photoreduction activity of BTOPAu with selected SACs systems and bismuth-based photocatalysts reported in the references. 26

Supplementary Table 4. CO_2 adsorption of BTO, BTOP, $BTOAu_3$ and BTOPAu. 27

Supplementary References **28**

Supplementary Figures

1. XRD patterns of the as-synthesized products

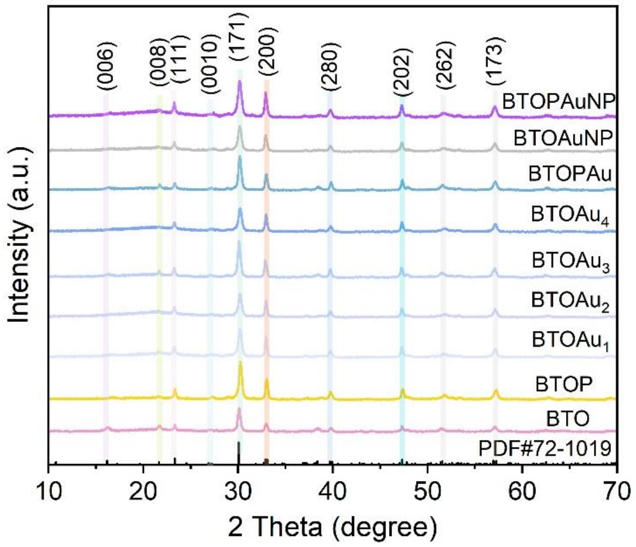

Supplementary Fig. 1 XRD patterns of BTO, BTOP, BTOAu$_X$ (X=1, 2, 3, 4), BTOPAu, BTOAuNP and BTOPAuNP.

The detailed information about the purity and crystallinity of the samples is obtained by the analysis of the XRD measurement. The diffraction peaks of BTO coincide with the standard data of $Bi_4Ti_3O_{12}$ (PDF#72-1019), indicating the good crystallinity. In addition, no other phases can be found in the series of BTO samples, indicating that the introduction of Au loading does not produce impurities or change the crystal structure. The Deposition Number 1631825 contains the supplementary crystallographic data for this paper. These data are provided free of charge by the joint Cambridge Crystallographic Data Centre and Fachinformationszentrum Karlsruhe.

2. SEM images of the as-synthesized products

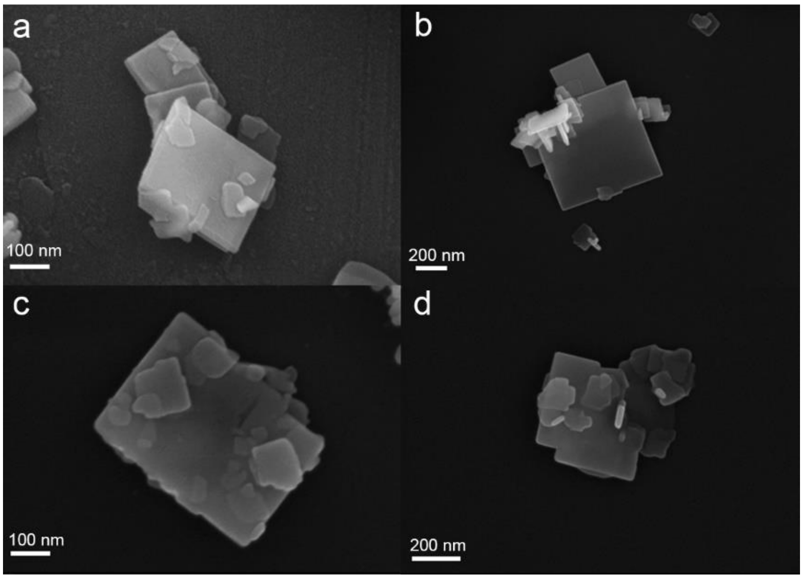

Supplementary Fig. 2 SEM images of **a** BTO, **b** BTOP, **c** $BTOAu_3$ and **d** BTOPAu.

SEM images show that series of BTO samples possess a sheet-like morphology.

3. AFM image of BTOAuNP

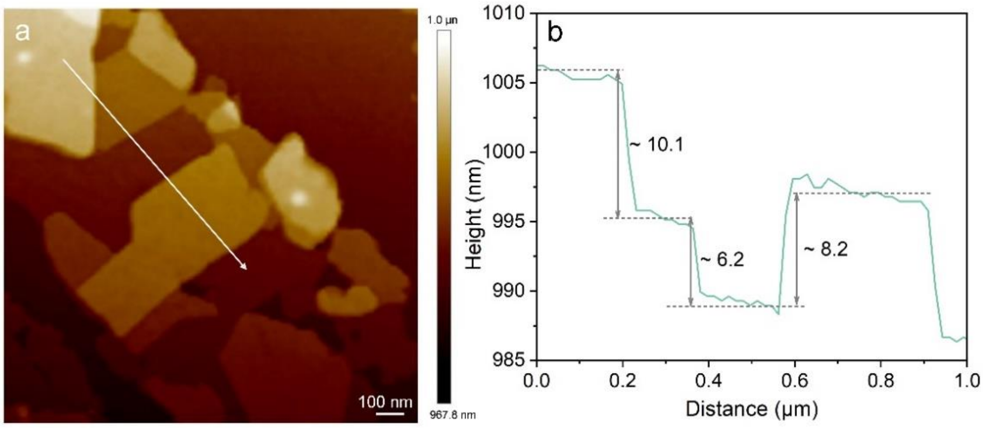

Supplementary Fig. 3 a AFM image and **b** corresponding curve (white narrow in **a**) of BTOPAu.

4. TEM images of BTOAuNP

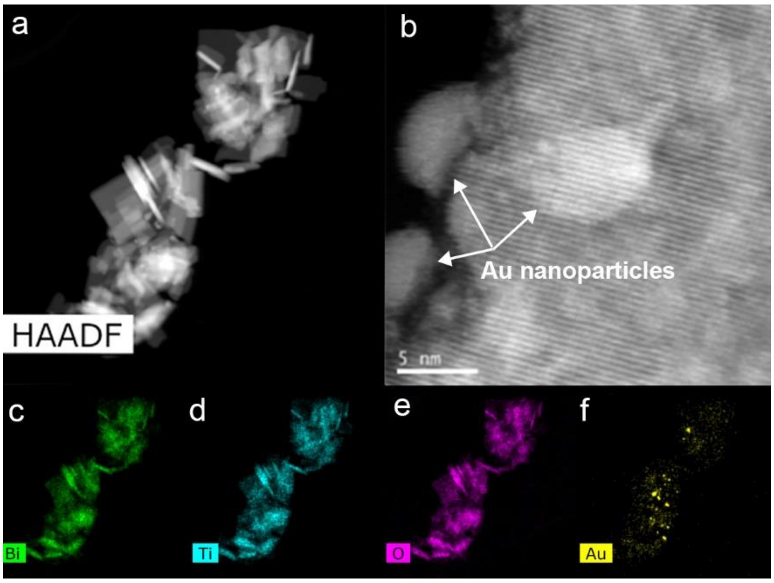

Supplementary Fig. 4 a, b TEM images and **c-f** element mapping of BTOAuNP.

TEM images show that Au are deposited on BTOAuNP surface as nanoparticles.

5. HAADF-STEM images and HAADF intensity profiles of $BTOAu_3$ and BTOPAu

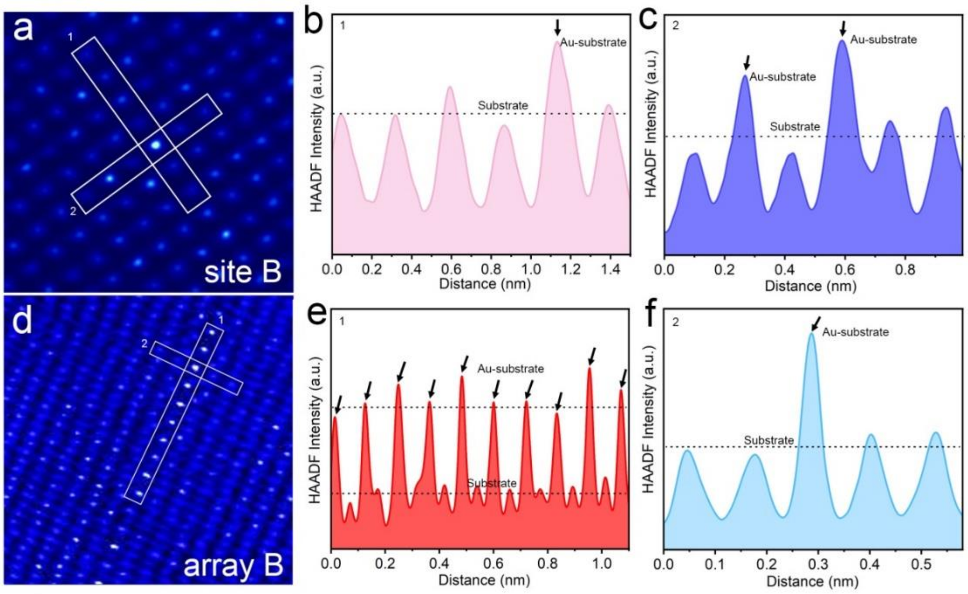

Supplementary Fig. 5 a HAADF-STEM images of $BTOAu_3$. **b, c** Line scan HAADF intensity profile of isolated sites (site B) as distance. **d** HAADF-STEM images of BTOPAu. **e, f** Line scan HAADF intensity profile of isolated sites (array B) as distance.

The two-dimension HAADF intensity of bright dot (site B) shows obviously stronger intensity compared to surrounding atoms, indicating the formation of Au i-SAs on the surface of BTO. And two-dimension HAADF intensity of bright dots (array B) exhibits two intensity levels along direction 1, where the low intensity level corresponds to Bi/Ti atoms and the high level belongs to the periodic Au atoms loading. The HAADF intensity also show only a high intensity bright dot along direction 2, which confirms the 1D Au SA arrays structure.

6. EXAFS data fitting analysis

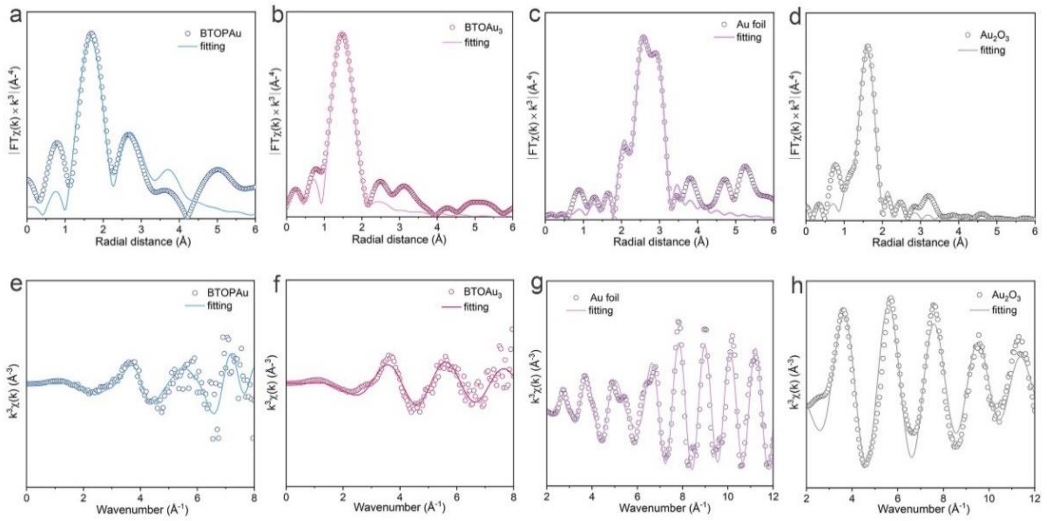

Supplementary Fig. 6 Fitting results of **a-d** the k^3-weighted FT spectrum in R space and **e-h** $k^3\chi$ data in K space of BTOPAu, $BTOAu_3$ and references at Au L_3-edge.

7. XPS spectra of the as-synthesized products

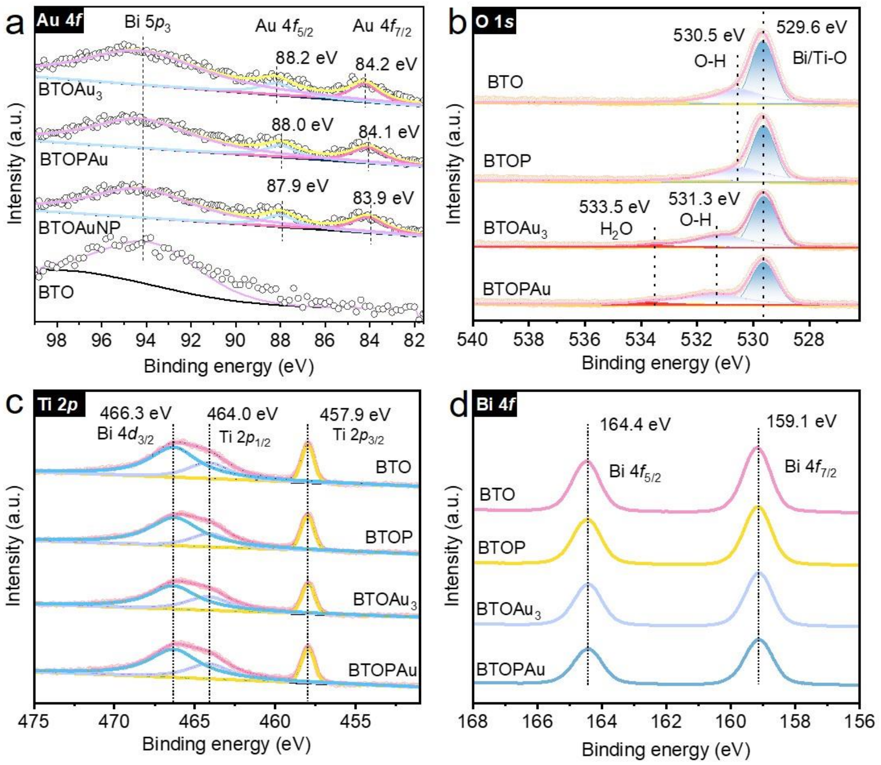

Supplementary Fig. 7 XPS spectra: **a** Au 4*f* of $BTOAu_3$, BTOPAu and BTOAuNP, **b** O 1*s*, **c** Ti 2*p*, **d** Bi 4*f* of BTO, BTOP, $BTOAu_3$ and BTOPAu.

XPS spectra of Bi 4*f* and Ti 2*p* of BTO, BTOP, $BTOAu_3$ and BTOPAu have no obvious difference and O 1*s* of $BTOAu_3$ and BTOPAu differs from that of BTO and BTOP, which indicates that Au SA successfully anchors on BTO surface and Au atoms coordinate with O atoms. And a peak at 533.5 eV in O 1*s* spectra can be indexed as peak of H_2O adsorption, which shows that H_2O favors to adsorb on $BTOAu_3$ and BTOPAu surface, and uncovers that Au SAs loading provides advantage for CO_2 activation.

8. Raman spectra of the as-synthesized products

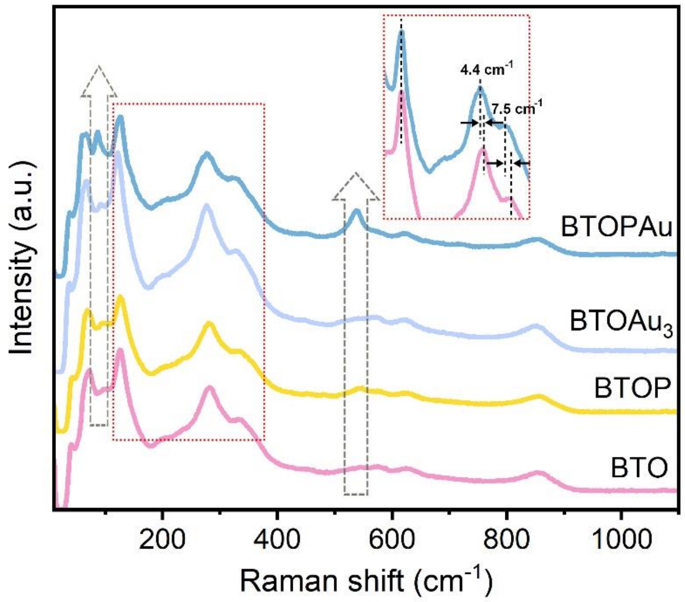

Supplementary Fig. 8 Raman spectra of BTO, BTOP, $BTOAu_3$ and BTOPAu.

9. HAADF-STEM images of metals loaded on BTO and BTOP.

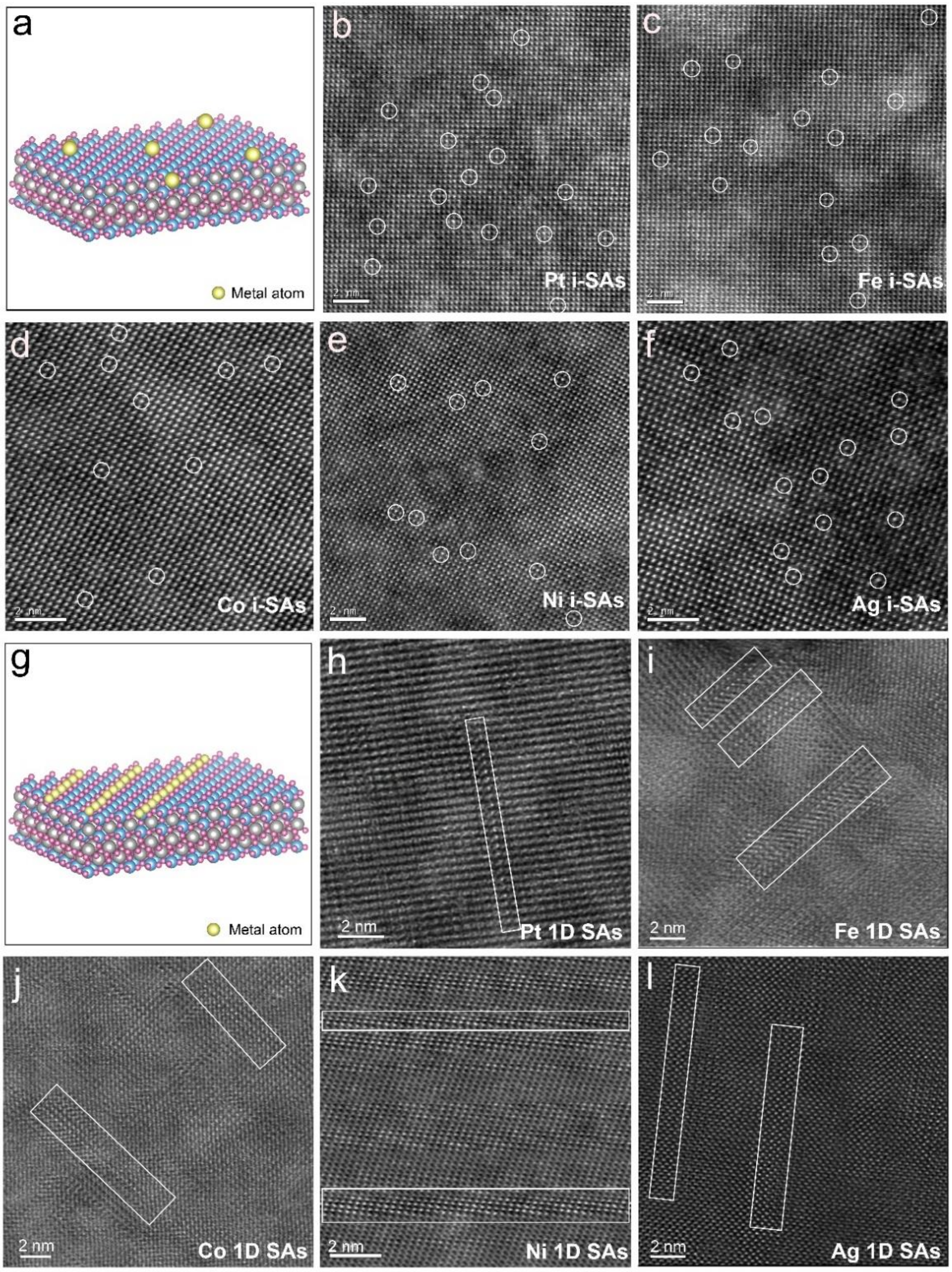

Supplementary Fig. 9 Configuration of metal SAs loaded on BTO and BTOP. Schematic diagram of metals loaded on **a** BTO and **g** BTOP. HAADF-STEM images of metals (Pt, Fe, Co, Ni and Ag) loaded on **b-f** BTO and **h-l** BTOP.

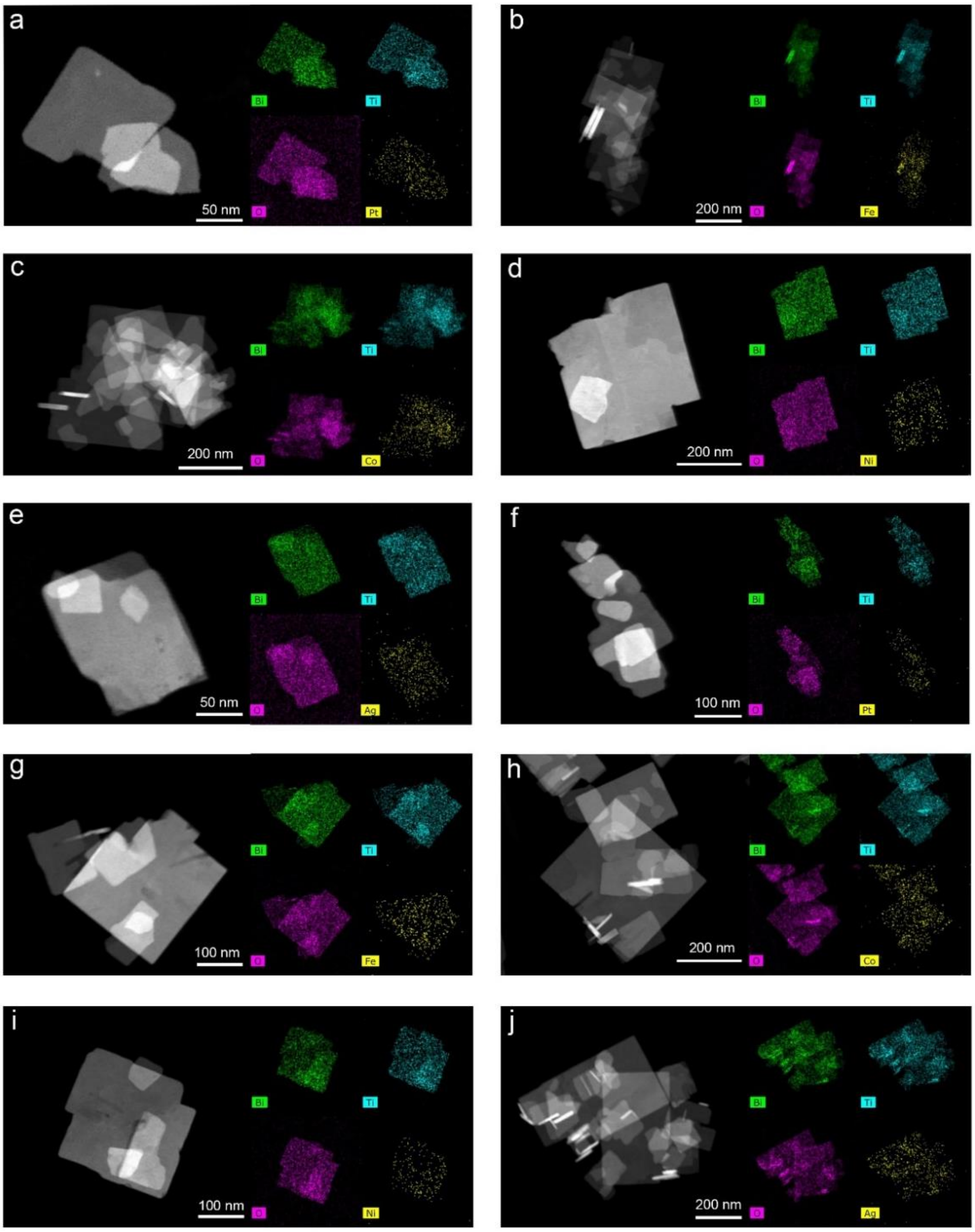

Supplementary Fig. 10 Element mapping corresponding to the metal SAs (Pt, Fe, Co, Ni and Ag) loaded on **a-e** BTO and **f-j** BTOP.

10. CO production over BTOAuNP and BTOPAuNP.

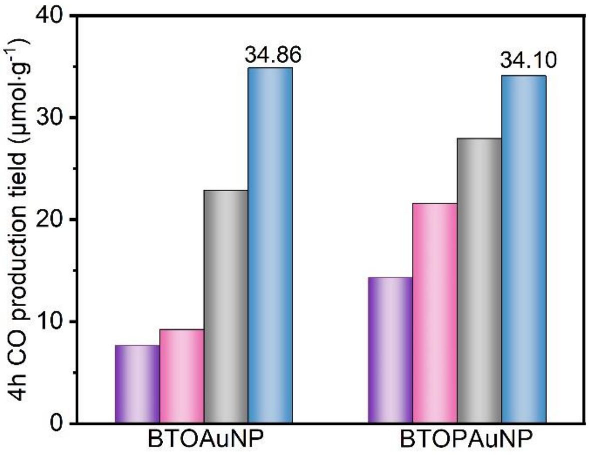

Supplementary Fig. 11 CO production from CO_2 photoreduction over BTOAuNP and BTOPAuNP for 4 h under simulated solar light.

11. Cycling test

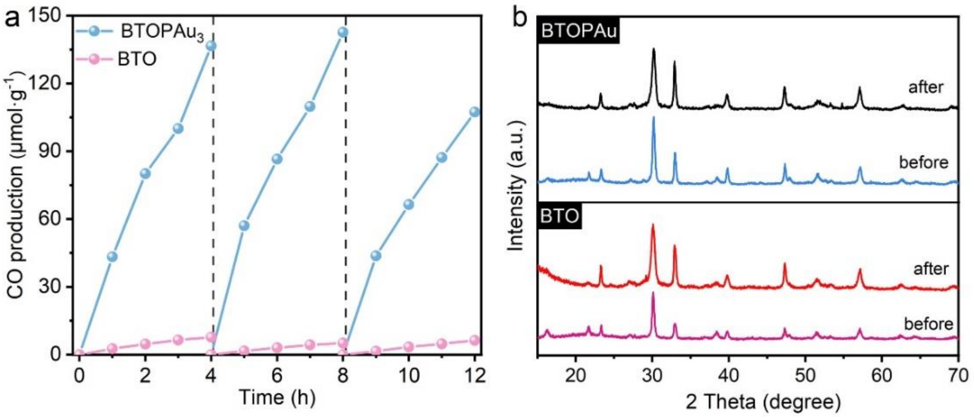

Supplementary Fig. 12 a Cycling test of photoreduction CO_2 reduction into CO over BTO and BTOPAu. **b** XRD patterns of BTO and BTOPAu before and after reaction.

12. Mott-Schottky plots of as-synthesized products

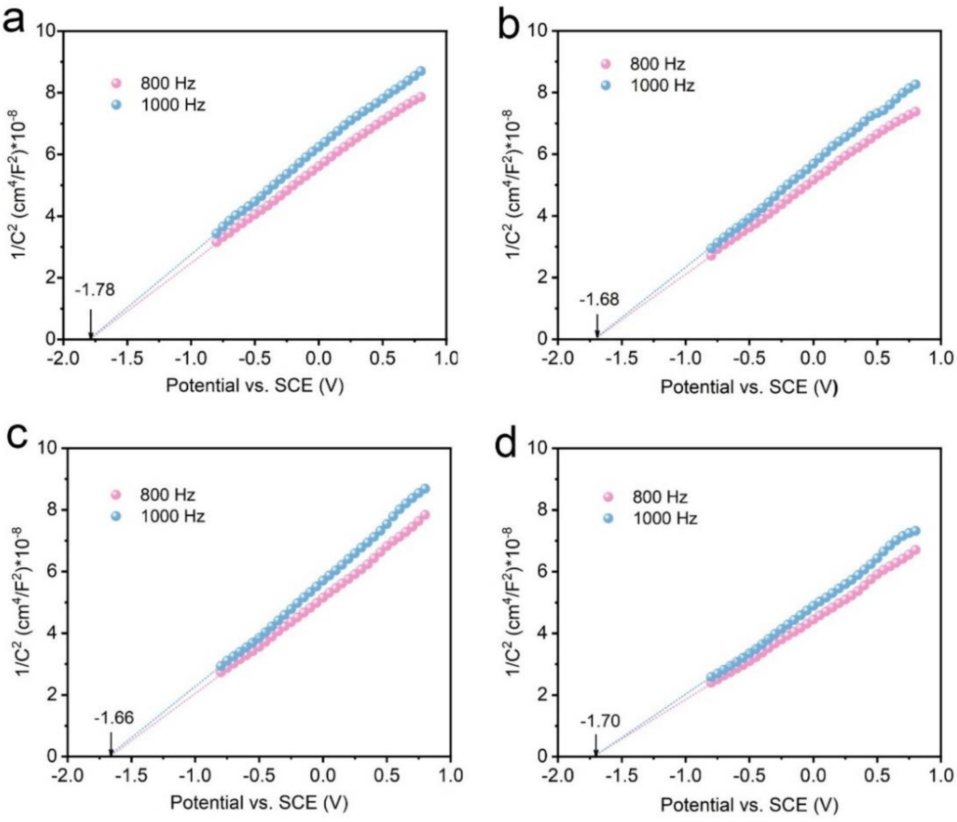

Supplementary Fig. 13 Mott-Schottky plots of **a** BTO, **b** BTOP, **c** $BTOAu_3$ and **d** BTOPAu.

Mott-Schottky plots show that the CB positions of BTO, BTOP, $BTOAu_3$ and BTOPAu are approximately -1.54, -1.44, -1.42 and -1.46 V vs. normal hydrogen electrode (NHE), respectively.

The translation formula from saturated calomel electrode (SCE) to NHE is as followed:[1]

$$E_{(\mathrm{vs\ NHE})} = E_{(\mathrm{vs\ SCE})} + 0.24\ \mathrm{V} \quad (1)$$

13. DRS spectra and energy band diagram of as-synthesized products

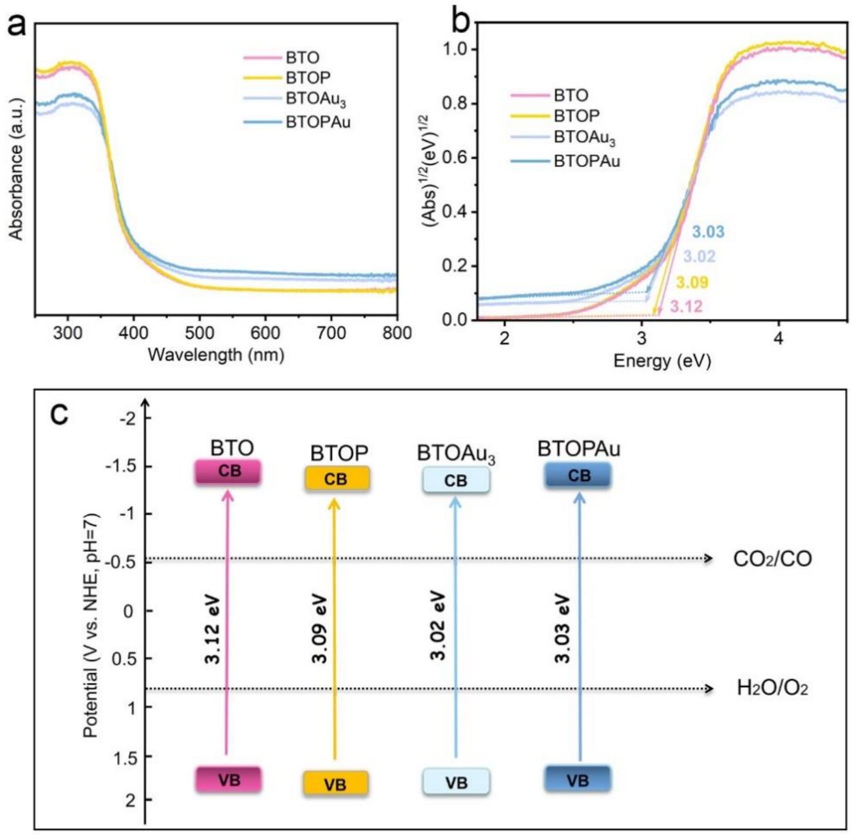

Supplementary Fig. 14 a DRS spectra, **b** bandgap and **c** energy band diagram of BTO, BTOP, $BTOAu_3$ and BTOPAu (the difference value between Mott-Schottky plots results and conduction band has been considered as 0.2 V).

14. PL spectra of as-synthesized products

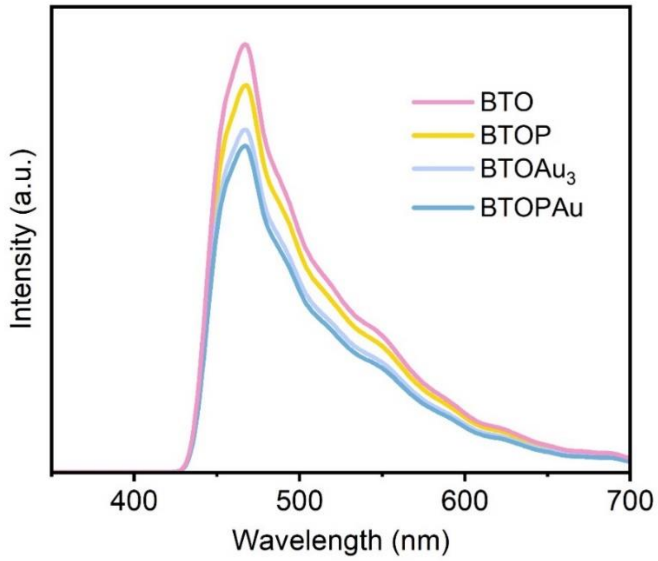

Supplementary Fig. 15 PL spectra of BTO, BTOP, $BTOAu_3$ and BTOPAu.

15. Surface charge density of BTO and BTOPAu

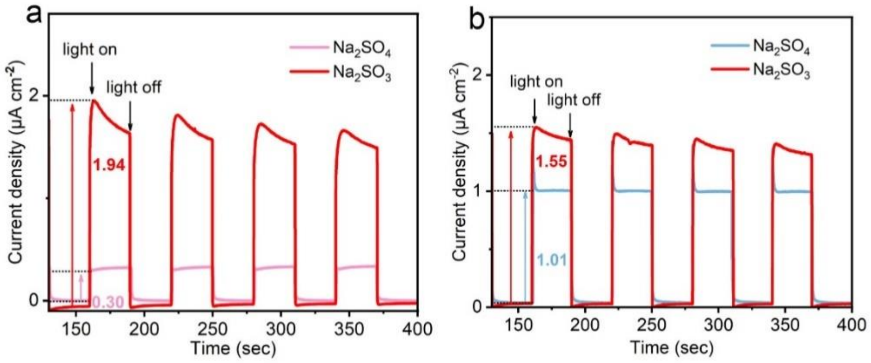

Supplementary Fig. 16 Transient photocurrent responses of **a** BTO and **b** BTOPAu with 0.1 M Na_2SO_4 electrolyte and, 0.1 M Na_2SO_4 and 0.1 M Na_2SO_3 electrolyte.

Na_2SO_3, as holes scavenger, was added into electrolyte (0.1 M Na_2SO_4) to quantitative calculate the surface charge transfer efficiency (η_{trans}).[2] The photocurrent can be defined by the following equation (2):

$$J_{H_2O} = J_{max} \cdot \eta_{abs} \cdot \eta_{sep} \cdot \eta_{trans} \quad (2)$$

The surface charge transfer is very fast and the η_{trans} can be considered as 100% when adding holes scavenger SO_3^{2-} to electrolyte,. The photocurrent can be voiced as equation (3):

$$J_{SO_3{}^{2-}} = J_{max} \cdot \eta_{abs} \cdot \eta_{sep} \quad (3)$$

Where, J_{max}, η_{abs}, η_{sep} are unchanged for both J_{H_2O} and $J_{SO_3{}^{2-}}$. Therefore, the η_{trans} can be counted as followed equation (4):

$$\eta_{trans} = J_{H_2O}/J_{SO_3{}^{2-}} \quad (4)$$

After calculation, the η_{trans} of BTO and BTOPAu is 15% and 67%, respectively, which indicates that Au SAs arrays anchoring can promote surface charge separation.

16. Relax structures of DFT calculation

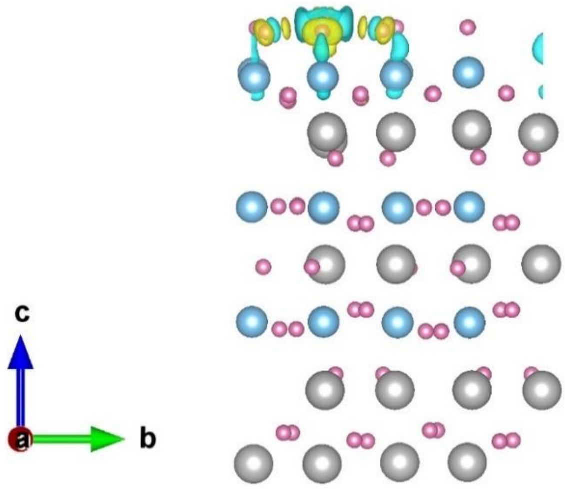

Supplementary Fig. 17 Charge difference of $BTOAu_3$.

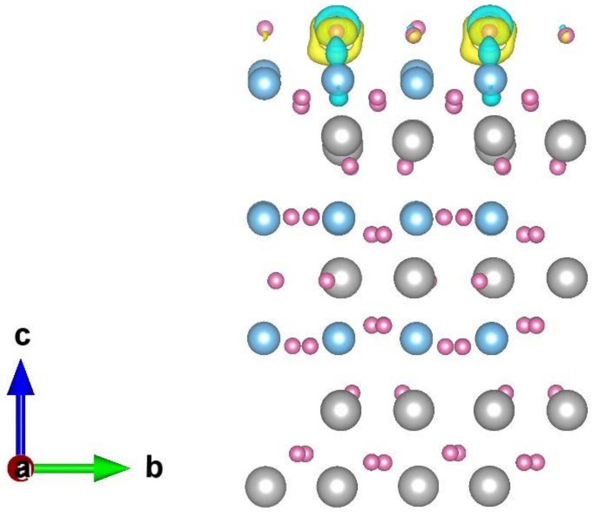

Supplementary Fig. 18 Charge difference of BTOPAu.

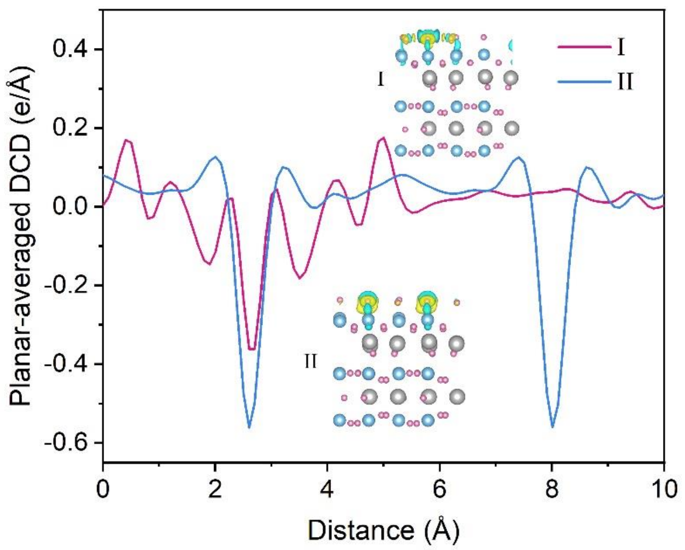

Supplementary Fig. 19 Planer charge difference curves of $BTOAu_3$ and BTOPAu.

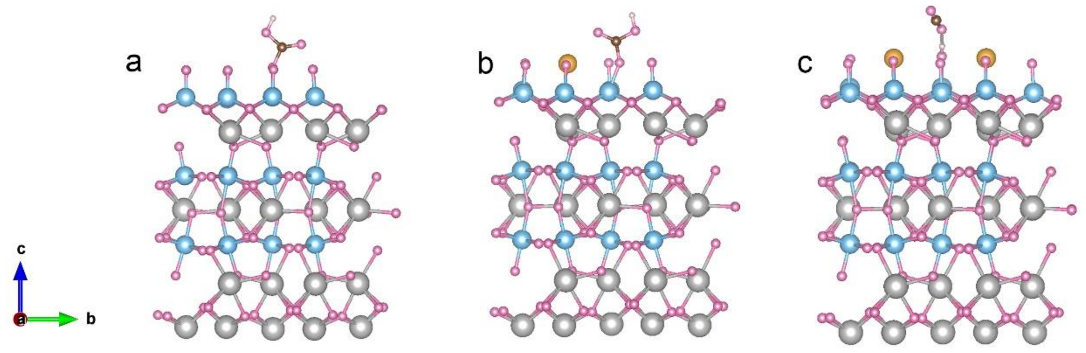

Supplementary Fig. 20 Relax structures of COOH absorbed on **a** BTO, **b** $BTOAu_3$ and **c** BTOPAu.

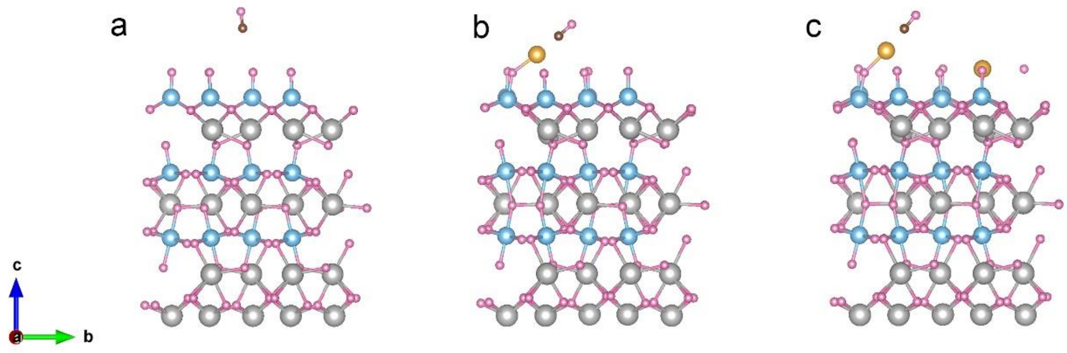

Supplementary Fig. 21 Relax structures of CO absorbed on **a** BTO, **b** $BTOAu_3$ and **c** BTOPAu.

Supplementary Tables

Supplementary Table 1. Au content based on ICP results.

Samples	Theoretical value (ppm)	Tested value (ppm)	Au loading (wt%)
$BTOAu_1$	7.5	3.15	0.11
$BTOAu_2$	15	4.56	0.15
$BTOAu_3$	30	7.98	0.27
$BTOAu_4$	60	13.20	0.44
BTOPAu	30	8.87	0.3

For ICP measure: 30 mg sample has been dissolved into 5 mL nitric acid for 24 h, then the solution has been purified using 0.45 μm filter. The obtained solution has been filled up to 10 mL for ICP test.

Supplementary Table 2. EXAFS fitting parameters at the Au L_3-edge for samples.

Sample	Shell	CN^a	R(Å)^b	σ^2(Å^2)^c	ΔE_0(eV)^d	R factor
Au-foil	Au-Au	8*	2.845±0.019	0.0151	6.7	0.0032
	Au-Au	6*	2.855±0.009	0.0050	3.1	
Au_2O_3	Au-O	4.1±0.5	1.986±0.001	0.0054	10.6	0.0124
$BTOAu_3$	Au-O	3.8±0.2	1.964±0.007	0.0074	7.4	0.0098
BTOPAu	Au-O	1.9±0.1	2.035±0.001	0.0016	13.1	0.0180
	Au-Au	4.4±0.9	3.524±0.001	0.0010	3.2	

$^a CN$, coordination number; $^b R$, the distance to the neighboring atom; $^c \sigma^2$, the Mean Square Relative Displacement (MSRD); $^d \Delta E_0$, inner potential correction; R factor indicates the goodness of the fit. $S0^2$ was fixed to 0.856, according to the experimental EXAFS fit of Au foil by fixing CN as the known crystallographic value. * This value was fixed during EXAFS fitting, based on the known structure of Au. Fitting range: $3.0 \leq k$ (/Å) ≤ 12.0 and $1.0 \leq R$ (Å) ≤ 3.3 (Au foil); $3.0 \leq k$ (/Å) ≤ 12.0 and $1.0 \leq R$ (Å) ≤ 2.0 (Au_2O_3); $1.5 \leq k$ (/Å) ≤ 8.0 and $1.0 \leq R$ (Å) ≤ 2.2 ($BTOAu_3$); $2.5 \leq k$ (/Å) ≤ 7.8 and $1.1 \leq R$ (Å) ≤ 3.2 (BTOPAu). A reasonable range of EXAFS fitting parameters: $0.700 < S_0^2 < 1.000$; $CN > 0$; $\sigma^2 > 0$ Å^2; $|\Delta E_0| < 15$ eV; R factor < 0.02.

Supplementary Table 3. Comparison of the CO_2 photoreduction activity of BTOPAu with selected SACs systems and bismuth-based photocatalysts reported in the references.

Photocatalysts	Light source	Solvent	Production ($\mu mol \cdot g^{-1} \cdot h^{-1}$)	Ref.
BTOPAu	300 W Xe lamp	Gas-solid	CO: 34.15	This work
$Bi_4O_5I_2$–Fe30	300 W Xe lamp	Water	CO: 23.77; CH_4: 4.98	3
Single Ni sites on g-C_3N_4	300 W Xe lamp	Water	CO: 22.1; CH_4: 8.7	4
Ni-SA-x/ZrO_2	300 W Xe lamp	Water	CO: 11.8	5
Mo-COF	300 W xenon lamp ($\lambda \geq 420$ nm)	Water	CO: 6.19	6
Cu SAs/UiO-66-NH_2	300 W Xe lamp with a cut-off filter of 400 nm	Water and TEOA	Methanol: 5.33; Ethanol: 4.22	7
Pt-SA/CTF-1	visible light ($\lambda \geq$ 420 nm)	Water and TEA	CO: 4.75	8
Fe SA on g-C_3N_4	300 W Xe lamp	Water	CO: 0.51	9
$SrBi_4Ti_4O_{15}$	300 W Xe lamp	Gas-solid	CO: 19.8	10
Bi_2MoO_6	300 W Xe lamp	Gas-solid	CO: 14.38	11
$BiVO_4$/$Bi_4Ti_3O_{12}$	300 W Xe lamp	Gas-solid	CO: 13.29	12
$SrBi_2Nb_2O_9$	300 W Xe lamp	Gas-solid	CH_4: 8.75	13

Supplementary Table 4. CO_2 adsorption of BTO, BTOP, $BTOAu_3$ and BTOPAu.

Samples	Press. Tolerance	Bath Temp	Volume @ STP
BTO	0.100	298.0 K	0.9216
BTOP	0.100	298.0 K	1.0448
$BTOAu_3$	0.100	298.0 K	2.3172
BTOPAu	0.100	298.0 K	2.7390

Supplementary References

1 Y. Shi *et al.* Enhanced photocatalytic hydrogen production activity of CdS coated with Zn-anchored carbon layer. *Chem. Eng. J.* **393**, 124751 (2020).

2 C. Y. Liu *et al*. Intermediate-mediated strategy to horn-like hollow mesoporous ultrathin g-C_3N_4 tube with spatial anisotropic charge separation for superior photocatalytic H_2 evolution. *Nano Energy* **41**, 738-748 (2017).

3 X. L. Jin *et al*. Single-atom fe triggers superb CO_2 photoreduction on a bismuth-rich catalyst. *ACS Mater. Lett.* **3**, 364-371 (2021).

4 Y. Y. Wang *et al*. Construction of six-oxygen-coordinated single Ni sites on g-C_3N_4 with boron-oxo species for photocatalytic water-activation-induced CO_2 reduction. *Adv. Mater.* **33**, 2105482 (2021).

5 X. Y. Xiong *et al*. Photocatalytic CO_2 reduction to CO over Ni single atoms supported on defect-rich zirconia. *Adv. Energy Mater.* **10**, 2002928 (2020).

6 M. P. Kou et al. Photocatalytic CO_2 conversion over single-atom MoN_2 sites of covalent organic framework. *Appl. Catal. B* **291**, 120146 (2021).

7 G. Wang *et al*. Photoinduction of Cu single atoms decorated on UiO-66-NH_2 for enhanced photocatalytic reduction of CO_2 to liquid fuels. *J. Am. Chem. Soc.* **142**, 19339-19345 (2020).

8 G. C. Huang *et al*. Platinum single-atoms anchored covalent triazine framework for efficient photoreduction of CO_2 to CH_4. *Chem. Eng. J.* **427**, 131018 (2022).

9 Z. Y. Zhao *et al.* An insight into the reaction mechanism of CO_2 photoreduction catalyzed by atomically dispersed Fe atoms supported on graphitic carbon nitride. *Phys. Chem. Chem. Phys.* **23**, 4690-4699 (2021).

10 S. C. Tu *et al*. Ferroelectric polarization promoted bulk charge separation for highly efficient CO_2 photoreduction of $SrBi_4Ti_4O_{15}$. *Nano Energy* **56**, 840-850 (2019).

11 S. G. Li *et al*. Ferroelectric polarization and thin-layered structure synergistically promoting CO_2 photoreduction of Bi_2MoO_6. *J. Mater. Chem. A* **8**, 9268-9277 (2020).

12 X. Y. Wang *et al.* $BiVO_4$ /$Bi_4Ti_3O_{12}$ heterojunction enabling efficient photocatalytic reduction of CO_2 with H_2O to CH_3OH and CO. *Appl. Catal. B* **270**, 118876 (2020).

13 H. J. Yu *et al.* Synergy of ferroelectric polarization and oxygen vacancy to promote CO_2 photored*uction. Nat. Commun. **12**, 4594 (2021).*
